# Zinner Syndrome: The Diagnosis and Management of a Rare Urogenital Malformation

**DOI:** 10.1155/2024/1718485

**Published:** 2024-07-15

**Authors:** Lucinda Lau, Kishan S. Patel, Frank Santisi, Rebecca Germaine, Sunil Jeph

**Affiliations:** ^1^ Department of Radiology Cooper Medical School of Rowan University, Camden, New Jersey 08103, USA; ^2^ Department of Radiology Cooper University Hospital, Camden, New Jersey 08103, USA; ^3^ Department of Public Health George Washington University, Washington, DC 20052, USA

## Abstract

This case highlights an atypical but important consideration in young males presenting with persistent gastrointestinal and/or genitourinary symptoms. Zinner syndrome (ZS) develops from embryologic maldevelopment of the distal mesonephric duct, resulting in ejaculatory duct atresia with consequent obstruction of the seminal vesicle and concomitant ureteral bud malformation, leading to renal agenesis/dysplasia. The lack of distinct clinical symptoms makes ZS a difficult diagnosis to reach: Abdominal pain and dysuria are often mistaken for prostatitis or cystitis. However, the use of modern imaging modalities aids in establishing the diagnosis. Early identification of ZS may delay progression to infertility as the duct obstruction may not be as extensive, though further research is needed to establish this connection.

## 1. Introduction

Zinner syndrome (ZS) is a congenital abnormality associated with a variety of reproductive and renal system malformations. The etiology of ZS can be attributed to an error in embryogenesis during the first 4–13 weeks of fetal development [1]. Parallels can be made between ZS and congenital anomalies in females such as Müllerian agenesis or Herlyn–Werner–Wunderlich syndrome (HWWS). Like HWWS, which involves uterine and vaginal malformations due to abnormal Müllerian duct development, ZS arises from maldevelopment of the mesonephric duct, leading to ejaculatory duct obstruction, seminal vesicle issues, and renal agenesis/dysplasia [2].

During typical embryological development of the kidneys, the mesonephric stage is essential in establishing basic renal architecture. At approximately Week 5 of gestation, the mesonephric duct extends toward the cloaca. At this stage, the metanephric blastema will typically secrete glial cell-derived neurotrophic factor to induce outgrowth of the ureteric bud from the distal portion of the mesonephric duct to form the collecting system and its components, including glomeruli and tubules [3].

In ZS, the ureteric bud fails to properly contact the metanephric blastema, an essential step for the formation of the renal system. These developmental abnormalities lead to reproductive and renal system consequences—renal agenesis as well as ipsilateral atresia of the ejaculatory ducts. This results in the inability to drain fluid through the ejaculatory ducts leading to seminal vesicle cysts [4].

Currently, no definitive genetic or environmental component responsible for this congenital malformation has been identified. The rarity of the condition contributes to the lack of detailed information regarding its etiology with the estimated frequency of ZS being approximately 0.002% and approximately 200 reported cases in literature to date [5].

## 2. Clinical Presentation

A 25-year-old male with a significant history of left renal agenesis and hypothyroidism presented to a community hospital with multiple days of abdominal pain, constipation, and dysuria. Physical exam was significant for right lower leg edema, and the genitourinary exam was deferred.

## 3. Investigation

Initial computed tomography (CT) scans revealed a 10-cm heterogeneous pelvic soft tissue mass and right hydronephrosis in the setting of a normal creatinine level and PSA. The patient was then transferred to our major medical facility to escalate care.

Given the initial presentation, the following differential diagnoses were considered: prostatitis, prostate cancer, and iatrogenesis.

**Figure 1:**
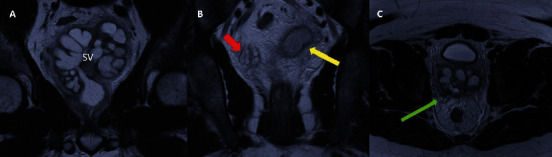
(A–C) A 25-year-old male presents with abdominal pain, dysuria, and constipation. (A) Coronal T2 MR image demonstrates a T2 hyperintense mass representing a dilated left seminal vesicle (SV). (B) Coronal T2 demonstrates a normal appearing right seminal vesicle (red arrow). The partially visualized left seminal vesicle is dilated (yellow arrow). (C) Axial T2 shows a normal appearing right ejaculatory duct (green arrow). The left ejaculatory duct is not visualized and demonstrates atresia of the ejaculatory duct. There is T2 hypointense signal layering within the dilated left seminal vesicle likely representing debris.

**Figure 2:**
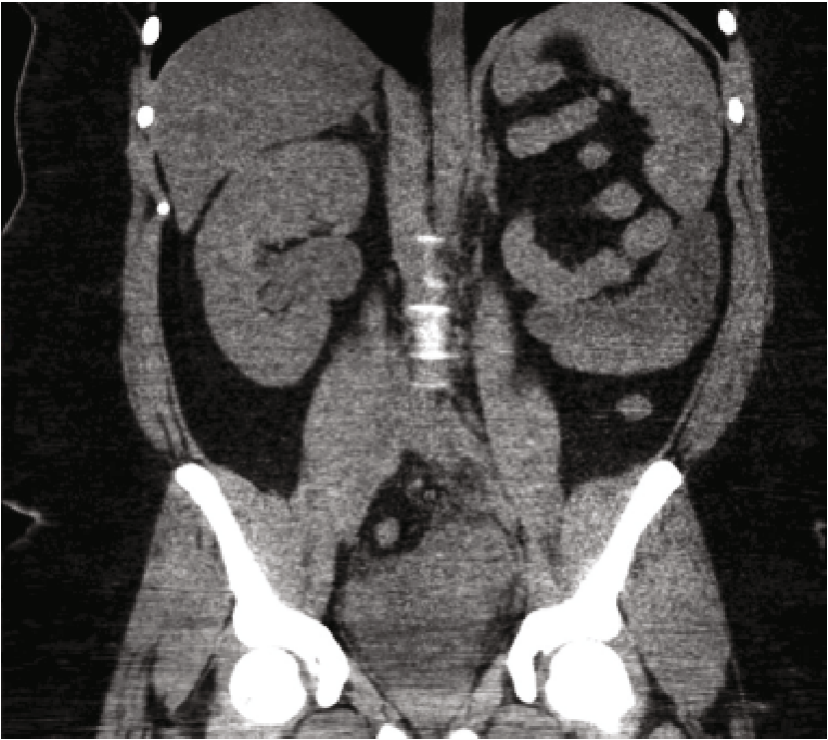
Coronal CT of the abdomen and pelvis without contrast demonstrates a normal appearing right kidney. The left kidney is not visualized which is consistent with left renal agenesis.

Pelvic magnetic resonance imaging (MRI) showed (a) a dilated left seminal vesicle cyst, (b) normal right seminal vesicle, and (c) atresia of the left ejaculatory duct (Figures 1(A), 1(B), and 1(C)). CT pelvis and abdomen demonstrated left renal agenesis (Figure 2). These findings confirmed the diagnosis of ZS.

## 4. Treatment and Follow-Up

The patient was discharged with a 2-week course of antibiotics and stool softeners. He was scheduled for a robot-assisted vesiculectomy and was instructed to follow-up with urology. Upon urology consultation, the patient was advised to obtain a semen analysis for infertility evaluation and sperm banking, if applicable, prior to treatment. Prior to the procedure, surgery counseled the patient again on the risk of infertility.

Approximately 2 weeks after discharge from our medical facility, the patient underwent surgery. During the robot-assisted vesiculectomy, four 8-mm robotic trocars were used along the right and left upper quadrants along with a 12-mm right upper quadrant AirSeal port. The patient was then placed in 22 degrees of Trendelenburg position, and the robot was docked from the side. The dilated pelvic mass was identified and carefully dissected along with the left vas deferens and seminal vesicle. Atrophy of the right seminal vesicle was confirmed, likely from compression by the left side cyst.

During his 1-month postoperative follow-up visit, the patient reported resolution of his pelvic pain and dysuria.

## 5. Discussion

ZS is typically asymptomatic until the second decade of life. Symptom onset is directly related to periods of increased sexual activity. The symptoms include nonspecific pain located in the scrotum, perineum, abdomen, or pelvis. Exacerbating factors normally are defecation and ejaculation. Additional symptoms include dysuria, urgency, and lower urinary tract infections. Due to the ejaculatory duct obstruction, approximately 45% of patients have some degree of infertility [6].

Initial imaging studies are ordered to evaluate unexplained symptoms such as abdominal pain. A CT scan of the abdomen and pelvis would show renal agenesis and an incompletely characterized pelvis mass. To further differentiate the pelvic mass, MRI can be performed which would demonstrate seminal vesicle cysts and ejaculatory duct atresia/obstruction. Therefore, the gold standard of diagnosis is an MRI pelvis to identify seminal vesicle cysts in the setting of ejaculatory duct obstruction [7].

### 5.1. Treatment Options and Outcomes

The management of ZS has evolved with the development of new technologies, surgical techniques, and data collection surrounding this rare condition. The choice of intervention also depends on whether the patient is symptomatic and the severity of these symptoms. As a result, careful clinical judgment must be made, considering both pain management and fertility, especially since ZS affects predominantly young males.

Less aggressive treatment methods are preferred for asymptomatic or mildly symptomatic ZS patients. Often, asymptomatic patients opt to forgo treatment and instead continue with regular follow-up appointments to monitor ZS progression. Those with mild symptoms or who prefer noninvasive treatment have the option to use *α*1-adrenoceptor antagonists to manage ejaculation pain and pelvic discomfort. A Uetani et al. study reported one case of ZS being successfully treated without recurrence of the seminal vesicle cyst over a 5-year follow-up period [8]. Still, more research is needed to properly evaluate the efficacy of this method in the long term. Mild symptoms may also be managed through transperineal or transurethral drainage of seminal vesicle cysts. The reality of this method, however, is that cysts often recur and symptoms are temporarily relieved, which requires repeated interventions [9].

More invasive ZS management, particularly surgical excision of the seminal vesicle cyst, is suggested only for symptomatic patients. This is considered the only treatment for ZS that is 100% effective. In the past, open surgery was performed via transabdominal or transperineal vesiculectomy. Complications of this method include potential damage to deep pelvic structures such as the bladder neck and wall, external urethral sphincter, and rectum. More recently, minimally invasive methods have become the gold standard for managing ZS [6, 9].

Laparoscopic surgery is the most common method used to surgically excise seminal vesicle cysts in ZS. Benefits of this modality include shorter hospital stay, reduced overall costs, minimal postoperative pain, and quicker recovery and return to normal daily activities. Robot-assisted seminal vesiculectomy has been gaining popularity as a treatment of choice for ZS as well. With similar benefits as laparoscopic surgery, robot-assisted surgery additionally allows for lower injury rates due to increased dexterity and advanced 3D capabilities that provide a more detailed dissection of deep pelvic structures [6, 9]. The limitation of this modality is not all medical facilities have access to this robotic platform.

In this case, many different factors contributed to the use of a more invasive surgical approach. Because he was symptomatic, robot-assisted surgical vesiculectomy was chosen, as it is a main modality to target pain relief in ZS. The biggest concern with this gold standard approach is that it does not address the concern of infertility, which is especially important in young patients. A technique to preserve fertility was used in the Valla et al. study, in which the team removed the majority of the seminal vesicle cyst in a 15-month-old boy laparoscopically, leaving only the cyst wall adjacent to the vas deferens. The goal was to determine whether early removal of the cyst could prevent long-term obstruction, possibly preserving fertility. Assessment of fertility, however, was not ascertained due to the patient being lost to follow-up [10]. Still, for those who are not concerned with maintaining fertility, which was the likely case for this patient, robot-assisted vesiculectomy is an ideal and effective treatment for symptomatic management of ZS.

Another important point of consideration is the size of the seminal vesicle cyst. Typically, patients with small cysts (< 5 cm) are asymptomatic. For these cases, it may be appropriate to consider less aggressive treatment methods [8]. Moderately sized cysts (5–12 cm) are associated with a more severe presentation including dysuria, pelvic pain, and infertility. With large cysts (> 12 cm), the immediate concern is for bladder/colon obstruction [9]. Our patient presented with a 10-cm pelvic mass, with symptoms consistent with both moderate and large-sized seminal vesicle cysts. As a result, it was important to choose the modality that would reverse these symptoms most effectively. Moreover, it is important for providers to consider how cyst size may contribute to azoospermia and infertility in ZS. The large size of this patient's left seminal vesicle cysts caused compression on the contralateral seminal vesicle and vas deferens, leading to atrophy. However, it is possible that early excision of a smaller cyst may have preserved contralateral structures, perhaps increasing the patient's chance of fertility. Still, further studies are needed to explore this connection.

Overall, the use of robot-assisted surgical excision proved effective in this case. At the 1-month follow-up visit, the patient reported a complete resolution of all symptoms except mild intermittent urinary retention that he stated he was not concerned with. He was able to return to work and daily activities within 20 days of his initial procedure and has not reported any new symptoms with our medical facility since his procedure a year ago.

### 5.2. Current Research and Future Directions

Though there is still more to be done to improve ZS outcomes, over the years, strides in the field have been made with diagnostic strategies and treatment approaches.

It is important to note that these interventions treat the physical symptoms and structural abnormalities of ZS. They fail, however, to address the infertility commonly associated with this diagnosis. Despite treatment in adulthood, azoospermia often persists in patients. One study proposed that infertility in ZS stems from long-lasting obstruction of the ejaculatory duct from accumulation of seminal fluid, which creates reactive oxidative species and reproductive toxicity [11]. Therefore, it would be logical to consider for future research how early treatment may mitigate infertility later in adulthood. A Valla et al. study attempted to treat ZS with laparoscopic vesiculectomy to preserve fertility in a 15-month-old boy. Unfortunately, the child was lost to follow up, so fertility status was not ascertained [10]. This attempt, though unsuccessful, is an important first step for future research in the maintenance of fertility in ZS.

## 6. Learning Points

• ZS should be considered in patients with nonspecific genitourinary symptoms exacerbated by periods of increased sexual activity, ejaculation, and defecation.
• Diagnosis can only be made through imaging by identifying a classic symptomatic triad: ejaculatory duct dysfunction, ipsilateral renal agenesis, and seminal vesicle cyst.
• Surgical excision of the seminal vesicle cyst is the gold standard for symptomatic patients.
• Infertility counseling is crucial to consider for this young patient population.
